# Describing Energy Expenditure in Children with a Chronic Disease: A Systematic Review

**DOI:** 10.1016/j.advnut.2024.100198

**Published:** 2024-03-01

**Authors:** Bethany Luo, Zoe E. Davidson, Katie O’Brien, Evelyn Volders, Jeffrey Lu, Kali Dunlea, Matisse Lazzari, Natassja Billich, Kay Nguo

**Affiliations:** 1 Department of Nutrition, Dietetics and Foods, Monash University, Melbourne, Victoria, Australia; 2 Murdoch Children’s Research Institute, Melbourne, Victoria, Australia

**Keywords:** systematic review, energy expenditure, children, indirect calorimetry, doubly labeled water

## Abstract

Understanding energy expenditure in children with chronic disease is critical due to the impact on energy homeostasis and growth. This systematic review aimed to describe available literature of resting (REE) and total energy expenditure (TEE) in children with chronic disease measured by gold-standard methods of indirect calorimetry (IC) and doubly labeled water (DLW), respectively. A literature search was conducted using OVID Medline, Embase, CINAHL Plus, Cochrane, and Scopus until July 2023. Studies were included if the mean age of the participants was ≤18 y, participants had a chronic disease, and measurement of REE or TEE was conducted using IC or DLW, respectively. Studies investigating energy expenditure in premature infants, patients with acute illness, and intensive care patients were excluded. The primary outcomes were the type of data (REE, TEE) obtained and REE/TEE stratified by disease group. In total, 271 studies across 24 chronic conditions were identified. Over 60% of retrieved studies were published >10 y ago and conducted on relatively small population sizes (*n* range = 1–398). Most studies obtained REE samples (82%) rather than that of TEE (8%), with very few exploring both samples (10%). There was variability in the difference in energy expenditure in children with chronic disease compared with that of healthy control group across and within disease groups. Eighteen predictive energy equations were generated across the included studies. Quality assessment of the studies identified poor reporting of energy expenditure protocols, which may limit the validity of results. Current literature on energy expenditure in children with chronic disease, although extensive, reveals key future research opportunities. International collaboration and robust measurement of energy expenditure should be conducted to generate meaningful predictive energy equations to provide updated evidence that is reflective of emerging disease-modifying therapies.

This study was registered in PROSPERO as CRD42020204690.

**Statement of Significance**

This systematic review provides a novel and unique contribution to existing literature as it is the first, to our knowledge, to comprehensively summarize the available evidence on energy expenditure measured using gold-standard techniques in children with chronic diseases. The inclusion of our extensive data extraction as supplementary information will serve as a valuable resource for clinicians and nutrition researchers to easily identify the available literature on energy expenditure in specific disease groups.

## Introduction

Childhood and adolescence are pivotal periods for the provision of appropriate nutrition for optimal growth and development [1]. Children and adolescents (“children”) with chronic diseases are a heterogenous group, with significant variation in their nutritional needs that differs from those of healthy populations. Chronic diseases in childhood can be defined using 4 criteria: *1*) the disease occurs in children aged 0–18 y; *2*) the diagnosis is based on medical scientific knowledge established via reproducible and valid methods; *3*) the disease is not (yet) curable or, for mental health conditions, it is highly resistant to treatment; and *4*) the disease has been present for longer than 3 mo or there have been 3 or more occurrences during the past year [2]. Chronic disease in children can impact growth potential, lean and fat tissue, bone mineral content, physiologic stress, physical function, activity levels, and underlying factors that may disrupt normal energy homeostasis. This complex set of factors has the potential to increase or decrease resting energy expenditure (REE), total energy expenditure (TEE), and/or the energy cost of growth, which already varies across age groups and sex in typically developing and healthy children [2].

Children with chronic diseases are at increased risk of malnutrition including underweight, overweight, and obesity [[3], [4], [5]]. Among select chronic conditions, obesity may be as high as 50% [6]. Understanding energy expenditure in children with chronic diseases is essential to prevent malnutrition through tailored dietetic advice and to inform clinical practice guidelines. Populations of particular concern for precise knowledge of measured energy expenditure are those requiring enteral nutrition. For these children, intrinsic hunger and satiety signals may be overridden (e.g., with the provision of continuous feeds), and for these groups, knowledge of measured energy expenditure is required for accurate feed prescription to avoid under- or overfeeding. Precise knowledge of measured energy expenditure is also important for conditions for which there is evidence that demonstrates improved clinical outcomes with optimized nutritional status, e.g., improved lung function and survival in cystic fibrosis [7] and improved survival following childhood cancer [8].

Energy expenditure is considered the amount of energy used by the body for cellular, metabolic and mechanical work, and its measurement continues to be recommended as a key component of comprehensive clinical nutrition assessments [9,10]. REE is the energy required to support the body’s basic metabolic activity; indirect calorimetry (IC) is the gold standard for its measurement [9,11]. TEE is the amount of energy used by the body in a free-living state incorporating REE, activity, and growth; doubly labeled water (DLW) is considered the gold standard of TEE measurement [12]. In a clinical setting, nutritional demands are predominantly estimated using predictive equations. However, energy expenditure can be assessed more accurately via direct measurements to obtain REE and TEE. This is particularly true in the context of children with chronic diseases, in which discrepancies between estimated and measured requirements may be present.

Although there are systematic reviews that explore energy expenditure in healthy children and those who are critically ill [[13], [14], [15]], to our knowledge, there is currently no review that has described the available literature for children with chronic disease. The aim of this systematic review was to systematically synthesize the available literature of REE and TEE in children with chronic disease measured by IC and DLW, respectively.

## Methods

### Design

This systematic literature review was conducted according to the PRISMA 2020 guidelines [16]. A full protocol is available via PROSPERO (CRD42020204690).

### Eligibility criteria

A Population, Intervention, Comparison, Outcomes, and Study (PICOS) strategy was developed to define the inclusion and exclusion criteria (Table 1). Studies that met the following criteria were included in the review: the mean age of participants was ≤18 y; the study reported REE measured by IC or TEE measured by DLW; and participants met the definition of having a chronic disease [17]. Although Mokkink et al. [17] defined chronic disease as present for longer than 3 mo, for the purpose of this review, the definition timeline was extended to 12 mo because we wanted to capture chronic diseases with longer disruptions to energy expenditure that would impact growth and nutritional status. Studies were also required to be peer-reviewed with full text available in the English language. Observational studies such as cohort, cross-sectional, and case-control studies were included as well as experimental studies that measured energy expenditure prior to an intervention. Systematic literature reviews and meta-analyses were also eligible for this review if they addressed the same research question. This was completed to capture the full synthesis of the current literature available for chronic diseases. Any data from systematic reviews or meta-analyses found were preferred over individual studies and cross-checked against included studies to mitigate double-counting.

**TABLE 1:** Population, Intervention, Comparison, Outcomes, and Study criteria for inclusion and exclusion of studies

	Inclusion criteria	Exclusion criteria
Population	Mean age < 18 y Met the definition of a chronic disease	Premature infants Low birth weight infants Stand-alone malnutrition (underweight, overweight, or obesity)
Intervention	Not applicable	Not applicable
Comparison	Not applicable	Not applicable
Outcomes	Resting energy expenditure measured via indirect calorimetry Total energy expenditure measured via doubly labeled water	Energy expenditure collected by surrogate methods Sleeping energy expenditure
Study Design	Any study design Peer-reviewed English language	Conference abstracts Dissertations/theses Case reports

Studies were excluded if they only included premature infants or low birthweight infants as this is more representative of an acute stage and hence would not meet the definition of a chronic disease. Studies were also excluded if participants had malnutrition (underweight, overweight, or obesity) without any additional comorbidities that met the definition of chronic disease or if energy expenditure samples were measured during the critical illness phase, in an intensive care unit, or during other acute illness time periods because these do not align with the definition of chronic disease. However, if REE/TEE was collected during rehabilitation periods after an acute illness that were likely to continue for 1 y, such studies were included (for example, rehabilitation after a burn injury). Studies that only included samples of energy expenditure using surrogate methods of energy expenditure collection (e.g., predicted through bioelectrical impedance analysis or commercially wearable devices) were excluded. Studies only reporting sleeping energy expenditure such as in children with sleep apnea were excluded because energy expenditure is known to be lower during sleep than wake periods [18]; however, studies that reported REE in infants who were sleeping were included, as this may be the only feasible way to collect REE during infancy. Conference abstracts, dissertations/theses, and case reports were excluded.

### Search strategy

An initial literature search was performed on OVID Medline, Embase, CINAHL Plus, Cochrane, and Scopus between the 26 and 28 August, 2020 and updated on 2 December, 2021 and 29 July, 2023. Search strategies included Medical Subject Heading terms and text words (Supplemental File 1). Key studies meeting the review inclusion criteria were identified to test the validity of the search before final execution of the searches. Identified references were imported into EndNote (version X9.3.3) for the removal of duplicates. References were then imported into the Covidence systematic review software (Veritas Health Innovation; available at www.covidence.org) for screening and management of the review process.

### Study selection strategy

Titles and abstracts of the retrieved references from the initial database search were independently assessed by ≥2 authors (ZED, BL, JL, NB, KN). Following the title and abstract screening, full-text documents of the selected studies were retrieved and independently assessed for inclusion by 2 authors (ZED, BL, JL, NB, KN) with a reason for exclusion assigned for studies not meeting eligibility criteria. Conflicts arising during study selection were resolved by discussions and consensus between conflicting authors.

### Data extraction

Data extraction was completed by all authors using Microsoft Excel. The data extraction form was first developed and piloted by the research team to ensure reliability of the fields in the form. Information was extracted by all members of the research team and included country, study design and information, population characteristics at baseline, REE results, TEE results, and quality assessment. Due to the number of included studies, 10% of included studies were randomly selected, and the accuracy of the data extraction was cross-checked by an independent researcher.

### Quality assessment

Studies were critically appraised for quality according to the Joanna Briggs Institute (JBI) Checklist for Analytical Cross-Sectional Studies [19]. An additional quality assessment tool developed by Porter et al. [20] was also utilized to evaluate the protocols used and adherence to best practice for the measurement of IC and DLW. Both quality assessment tools were completed by 1 researcher. Ten percent of included studies were randomly selected, and the accuracy of the quality assessment was cross-checked by an independent researcher.

### Data synthesis

Due to the large and heterogenous body of evidence retrieved in the review, a descriptive approach was taken to map the available literature, including a narrative synthesis supported by data tables describing study characteristics of disease-specific population groups. Included studies were first classified into disease categories and subcategories by the research team. For example, cancer was the disease category and the types of cancer (acute leukemia, solid tumor, brain tumor, etc.) were the subcategories. If multiple disease diagnoses were present within a study, disease-specific energy expenditure measurement data was synthesized in the relevant disease category. This meant a single study containing multiple chronic disease diagnoses may have been included under >1 disease category in the data synthesis. In reporting of results, we refer to either “studies,” which relates to characteristics of the complete included studies, or to energy expenditure “samples,” which refers to the number of REE and/or TEE measurements within each disease category and/or subcategory. This meant a single study may have had multiple samples if it measured both REE and TEE. If a disease was not further stratified into subcategories, it was included in the overarching disease category only (e.g., if the studies only reported that it measured children with cancer, this was included in the cancer disease category but not a subcategory). Studies that included diagnoses outside of the identified disease categories or that included multiple, unstratified disease categories were placed in the “other” category. When available, control comparisons were included where the control group was children without identified disease (i.e., healthy controls). When a study used a disease group comparison, the data from that disease group was instead classified under the relevant disease category. Although not all studies had a control comparison, all samples were included to ensure completeness of information to be summarized for future research.

All energy expenditure data reported in kcal were converted to kJ by multiplying by 4.184. Age of the participants was converted to years for all studies and recorded to the nearest 0.1 y except when the age was <0.05 y. If mean age was not available but the individual age values were reported in the study, the mean was calculated by the authors. Energy expenditure was not reported in the summary tables due to the large age ranges within disease categories, potentially impacting the interpretation of the energy expenditure values due to variability in developmental stages and body size. Instead, detailed data extraction, stratified by disease type, for individual studies including reported REE and TEE values (kJ) and population characteristics are provided in Supplemental File 2 to serve as a comprehensive resource of available energy expenditure studies in children with chronic diseases.

The type of data (REE, TEE) obtained, and REE/TEE compared with control when available, stratified by disease group was the primary outcome used in the generation of the results. The main summary table describes, for each disease category and subcategory: total number of samples reporting REE, TEE, or REE + TEE; whether energy expenditure was the primary research question; range of publication years; range of number of participants; range of mean age; and whether an equation was generated. A secondary summary table describes REE and TEE samples compared with control when available by disease group. A table was also generated to describe the quality and reporting of energy expenditure measurement protocols in the included studies.

## Results

**FIGURE 1:**
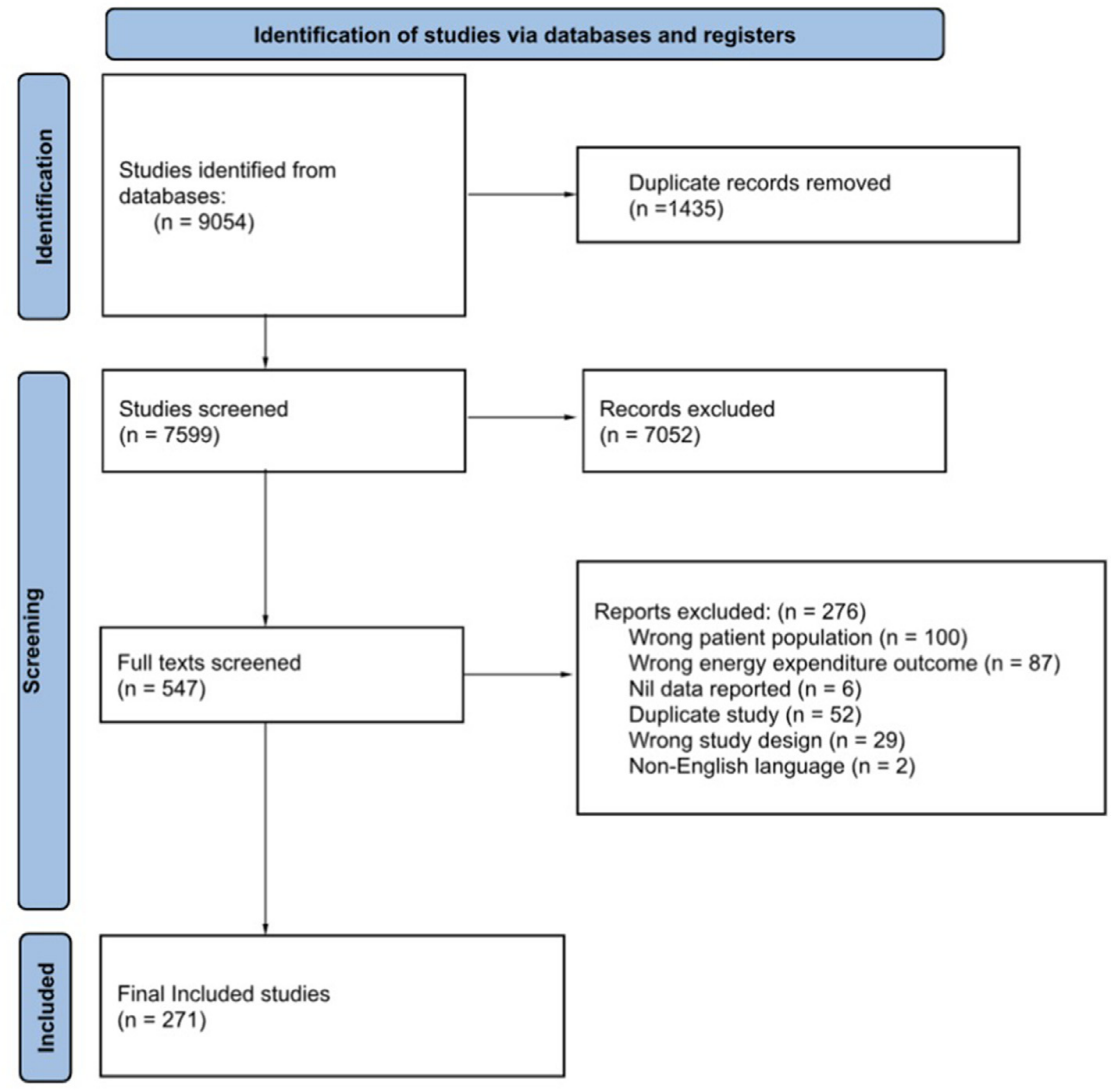
PRISMA 2020 flow diagram outlining the study selection process.

A total of 9054 studies were identified through the original and updated literature searches. After exclusion of duplicates and ineligible studies, 271 studies were retrieved. Within the included studies, there were 283 samples of REE and 52 samples of TEE across 24 disease categories. Figure 1 demonstrates the literature review process using the PRISMA flow diagram. Table 2 provides a summary of the included studies stratified by disease category and subcategory. Detailed information regarding study and participant characteristics, reported REE and TEE values, comparison with controls, and generated equations can be found in Supplemental File 2, stratified by disease categories.

**TABLE 2:** Number and characteristics of studies reporting samples of resting and/or total energy expenditure measured via indirect calorimetry and doubly labeled water in children with chronic disease stratified by chronic disease categories and subcategories, respectively

Disease category	EE samples (*k*)	REE samples *k* (%)	TEE samples *k* (%)	REE + TEE samples *k* (%)	EE as the primary research question *k* (%)	Publication y, range	Participant numbers, range	Age (y), range	Equations generated *k* (%)
Cancer	21	18 (86)	2 (10)	1 (5)	19 (90)	1991–2020	6–55	1.4–17.9	0 (0)
Acute leukemia	6	6 (100)	0 (0)	0 (0)	6 (100)	1997–2020	15–39	6.2–14.6	0 (0)
Solid tumor	3	3 (100)	0 (0)	0 (0)	3 (100)	2001–2008	12–37	3.8–8.8	0 (0)
Brain tumor	1	1 (100)	0 (0)	0 (0)	0 (0)	2020	10	12.4	0 (0)
Myelodysplasia	1	0 (0)	0 (0)	1 (100)	1 (100)	1991	10	16.6–17.9	0 (0)
Diencephalic syndrome	1	1 (100)	0 (0)	0 (0)	1 (100)	2014	9	1.4	0 (0)
Cerebral palsy	20	13 (65)	3 (15)	4 (20)	17 (85)	1965–2022	5–61	3.7–18.6	2 (10)
Congenital heart disease	15	7 (47)	4 (27)	4 (27)	14 (93)	1994–2015	7–44	0.03–14.4	0 (0)
Craniopharyngioma	7	7 (100)	0 (0)	0 (0)	5 (71)	1999–2017	1–39	10–16.5	0 (0)
Cystic fibrosis	48	38 (79)	4 (8)	6 (13)	44 (92)	1988–2020	4–134	0.1–18.4	2 (4)
Dermatologic conditions	6	5 (83)	0 (0)	1 (17)	4 (67)	1988–2017	7–76	5.6–10.4	0 (0)
Burns	4	3 (75)	0 (0)	1 (25)	2 (50)	2005–2017	10–76	7.3–10.4	0 (0)
Ichthyosis	1	1 (100)	0 (0)	0 (0)	1 (100)	2004	10	8.3	0 (0)
Epidermolysis bullosa	1	1 (100)	0 (0)	0 (0)	1 (100)	1988	7	5.6	0 (0)
Neurodevelopmental disability	9	5 (56)	4 (44)	0 (0)	9 (100)	1964–2021	12–256	7.1–15.1	2 (22)
Diabetes mellitus	6	6 (100)	0 (0)	0 (0)	5 (83)	1988–2022	12–18	9.3–17.8	0 (0)
Eating disorders	10	10 (100)	0 (0)	0 (0)	8 (80)	1992–2020	7–181	14–18.8	2 (20)
Anorexia nervosa	9	9 (100)	0 (0)	0 (0)	7 (78)	1992–2020	7–39	14–17.1	2 (22)
Bulimia nervosa	3	3 (100)	0 (0)	0 (0)	3 (100)	1995–2020	11–32	16.4–18.8	1 (33)
Endocrine (other)	12	12 (100)	0 (0)	0 (0)	11 (92)	1999–2022	3–292	0.1–15.5^1^	0 (0)
Thyroid dysfunction	1	1 (100)	0 (0)	0 (0)	1 (100)	1999	7	0.1	0 (0)
Growth hormone deficiency	1	1 (100)	0 (0)	0 (0)	1 (100)	1999	11	17^2^	0 (0)
Pseudohypoparathyroidism	3	3 (100)	0 (0)	0 (0)	3 (100)	2013–2018	6–19	12.2–12.6	0 (0)
Thyroid hormone resistance	1	1 (100)	0 (0)	0 (0)	1 (100)	2019	27	5.8–15.5	0 (0)
Congenital hypopituitarism	1	1 (100)	0 (0)	0 (0)	1 (100)	2008	13	8^2^	0 (0)
Hypothalamic dysfunction	3	3 (100)	0 (0)	0 (0)	3 (100)	2022	19–292	10.8–12.3	0 (0)
Gastrointestinal (other)	3	3 (100)	0 (0)	0 (0)	3 (100)	2003–2016	11–26	4.2–6^3^	1 (33)
Genetic disorders (other)	15	11 (73)	3 (20)	1 (7)	13 (87)	1994–2023	3–52	0.01^4^–16.7	0 (0)
Down syndrome	6	3 (50)	2 (33)	1 (17)	6 (100)	1994–2023	8–46	0.01–15.5	0 (0)
Williams syndrome	1	1 (100)	0 (0)	0 (0)	1 (100)	1998	3	11.4	0 (0)
Turner syndrome	2	2 (100)	0 (0)	0 (0)	0 (0)	2007–2013	11–41	13.4–16.7	0 (0)
Osteogenesis imperfecta	1	1 (100)	0 (0)	0 (0)	1 (100)	2018	52	9^2^	0 (0)
Alagille syndrome	2	2 (100)	0 (0)	0 (0)	2 (100)	1999–2006	13–16	6.8–9.8	0 (0)
Temple syndrome	1	1 (100)	0 (0)	0 (0)	1 (100)	2023	13	6.4	0 (0)
Human immunodeficiency virus	6	4 (67)	0 (0)	2 (33)	6 (100)	1995–2010	6–180	0.4–8.3	0 (0)
Inflammatory bowel disease	17	16 (94)	0 (0)	1 (6)	12 (71)	1996–2023	10–73	11.9–17.6	1 (6)
Crohn’s disease	13	13 (100)	0 (0)	0 (0)	9 (69)	1996–2023	10–73	13.1–17.6	0 (0)
Ulcerative colitis	2	2 (100)	0 (0)	0 (0)	1 (50)	2009–2016	6–18	13.7^2^–15	0 (0)
Liver disease	8	7 (88)	0 (0)	1 (13)	8 (100)	1989–2019	11–56	1.0–14.1^5^	1 (13)
Nonalcoholic fatty liver disease	3	3 (100)	0 (0)	0 (0)	3 (100)	2006–2017	16–56	13.6–14.1	0 (0)
Extrahepatic biliary atresia	1	1 (100)	0 (0)	0 (0)	1 (100)	1989	11	1.5	0 (0)
End-stage liver disease	2	1 (50)	0 (0)	1 (50)	2 (100)	2003–2019	17–21	1.0–7.4	0 (0)
Intestinal failure–associated liver disease	1	1 (100)	0 (0)	0 (0)	1 (100)	2014	28	0.4^2^	1 (100)
Extrahepatic portal vein obstruction	1	1 (100)	0 (0)	0 (0)	1 (100)	1996	14	12.3	0 (0)
Metabolic disorders	13	12 (92)	0 (0)	1 (8)	11 (84)	1989–2023	2–30	5–17^6^	0 (0)
Urea cycle disorders	1	1 (100)	0 (0)	0 (0)	1 (100)	2019	15	9.1–10.1^7^	0 (0)
Propionic and methylmalonic acidemias	2	2 (100)	0 (0)	0 (0)	2 (100)	2000–2004	8–14	2.5–9.0^2^	0 (0)
Pantothenate kinase-associated neurodegeneration	1	1 (100)	0 (0)	0 (0)	1 (100)	2013	6	14	0 (0)
Long chain 3-hydroxyacyl-CoA dehydrogenase deficiency	1	1 (100)	0 (0)	0 (0)	0 (0)	2015	5	7.5	0 (0)
Gauchers disease	1	1 (100)	0 (0)	0 (0)	1 (100)	1989	2	10	0 (0)
Phenylketonuria	1	1 (100)	0 (0)	0 (0)	1 (100)	1995	30	9.6	0 (0)
Mitochondrial oxidative phosphorylation disorders	1	1 (100)	0 (0)	0 (0)	1 (100)	2016	22	13.2	0 (0)
GLUT1 deficiency	1	1 (100)	0 (0)	0 (0)	1 (100)	2023	15	5–15	0 (0)
Long chain acid oxidation disorder	1	0 (0)	0 (0)	1 (100)	1 (100)	2023	12	7–17	0 (0)
Neurologic (other)	16	14 (88)	2 (12)	0 (0)	15 (94)	1994–2023	9–162	4.8–17.5	2 (13)
Rett syndrome	3	2 (67)	1 (33)	1 (33)	3 (100)	1994–2011	9–15	6.2–9.5	0 (0)
Epilepsy	3	3 (100)	0 (0)	0 (0)	3 (100)	1994–2014	10–17	6–11.8	0 (0)
Narcolepsy	1	1 (100)	0 (0)	0 (0)	1 (100)	2016	34	11.9	0 (0)
Spina bifida	4	3 (75)	1 (25)	0 (0)	4 (100)	2003–2018	19–162	8.1–15.8	0 (0)
Spinal cord injury	2	2 (100)	0 (0)	0 (0)	2 (100)	2007	33–59	14.8–17.5	1 (50)
Adrenoleukodystrophy	1	1 (100)	0 (0)	0 (0)	1 (100)	2023	9	11.49	0 (0)
Neuromuscular diseases	21	20 (95)	0 (0)	1 (5)	18 (86)	1992–2023	4–122	3.5–17.3^8^	1 (5)
Spinal muscular atrophy	6	6 (100)	0 (0)	0 (0)	5 (83)	2014–2023	4–122	3.5–16^8^	1 (17)
Duchenne muscular dystrophy	9	8 (89)	0 (0)	1 (11)	8 (89)	1992–2015	5–96	7–17.3	0 (0)
Becker muscular dystrophy	1	1 (100)	0 (0)	0 (0)	1 (100)	2008	2	10.8	0 (0)
Prader-Willi syndrome	13	9 (69)	1 (8)	3 (23)	6 (46)	1993–2021	4–63	1.03–15	2 (15)
Renal	6	5 (83)	0 (0)	1 (17)	6 (100)	1992–2023	8–51	9.4–14.6	0 (0)
Respiratory (other)	8	7 (88)	1 (13)	0 (0)	7 (88)	1992–2016	11–81	0.7–9.3	0 (0)
Chronic lung disease		3 (100)	0 (0)	0 (0)	3 (100)	2006–2016	18–71	0.7–6.1	0 (0)
Obstructive sleep apnea		0 (0)	1 (100)	0 (0)	1 (100)	2001	11	5.7	0 (0)
Asthma	1	1 (100)	0 (0)	0 (0)	1 (100)	1992	34	8.3^2^	0 (0)
Rheumatic	3	3 (100)	0 (0)	0 (0)	3 (100)	1999–2016	10–33	11.6–13.4	0 (0)
Juvenile arthritis	2	2 (100)	0 (0)	0 (0)	2 (100)	1999–2010	10–33	11.7–13.4	0 (0)
Sickle cell anemia	20	19 (95)	0 (0)	1 (5)	19 (95)	1982–2007	5–41	4.1–18	2 (10)
Other	4	4 (100)	0 (0)	0 (0)	4 (100)	1999–2016	2–398	0.1–12.5	0 (0)

Values expressed as mean ± SD unless specified.

Abbreviations: *k*, number of studies; REE, resting energy expenditure; TEE, total energy expenditure. Percentages calculated as percent of total disease categories and subcategories, respectively.

1 Highest mean available, note median of 17.0 [62].

2 Median, no mean available.

3 Median age for Beghin et al., 2003 [63], mean not available.

4 Includes control data as results not separated out.

5 Lowest mean available, median 0.4 y Duro et al. [21].

6 Lowest mean used. Median age 2.5 y for Van Hagen et al., 2004 [64].

7 Range for 2 disease groups in study (both urea cycle disorders).

8 Lowest mean reported. Note actual lowest Bertoli et al. [22], 1.1 y (only median available).

### Disease categories

Twenty-four disease categories were identified. Only 5 disease categories reported 20 or more energy expenditure samples within the respective category. The number of studies (*k*) within each category were as follows: cystic fibrosis (*k* = 48), cancer (*k* = 21), neuromuscular disease (*k* = 21), cerebral palsy (*k* = 20), and sickle cell anemia (*k* = 20). Eight different disease categories had between 10 and 19 energy expenditure samples (inflammatory bowel disease [IBD; *k* = 17], neurological conditions [*k* = 16], congenital heart disease [*k* = 15], genetic disorders [*k* = 15], Prader-Willi syndrome [*k* = 13], metabolic disorders [*k* = 13], endocrine [*k* = 12], and eating disorders [*k* = 10]). In the remaining 11 disease categories, there were <10 energy expenditure samples per category. The “other” category included 4 studies: 1 unstratified mixed disease cohort and 3 stratified mixed disease cohorts with conditions outside of the 24 main disease categories.

### Study characteristics

Studies were predominantly published from developed countries: United States (*k* = 110; 41%), United Kingdom (*k* = 28; 10%), Australia (*k* = 19; 7%), Canada (*k* = 17; 7%), and France (*k* = 15; 6%). Eighty-six (32%) studies were published before 2000, 94 (35%) studies were published between 2000 and 2010, and 91 (34%) studies were published after 2010. All studies of congenital heart disease, HIV, and sickle cell anemia were published before 2010.

Of the included studies, 260 (96%) had <100 participants, whereas 253 (93%) had <50 participants. Only 7 disease categories had participant cohorts >100: cystic fibrosis (*n* = 236 [23]; *n* = 134 [24]); developmental disability (*n* = 256 [25]); eating disorders (*n* = 181 [26]); endocrine (*n* = 292 [27]); neurological conditions (*n* = 236 [23]; *n* = 131 [28]); neuromuscular disease (*n* = 122 [22]); and other, mixed, and unstratified cohort (*n* = 236 [23]; *n* = 398 [29]). Age of participants ranged from birth to 18 y. Participants with HIV (0.4–8.3 y), gastrointestinal (4.2–6 y), and respiratory (0.8–9.3 y) conditions had younger mean ages whereas those with renal (9.4–14.6 y), eating disorders (14–18.8 y), and IBD (11.9–17.6 y) had older mean ages. All other conditions covered a wide range of ages.

### REE and TEE samples

**FIGURE 2:**
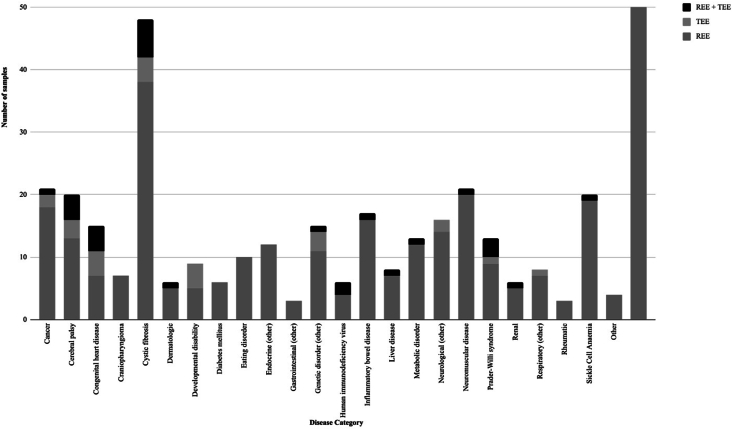
Number and type of studies reporting samples of measured resting and/or total energy expenditure via indirect calorimetry and doubly labeled water in children with chronic disease per disease category, respectively. REE, resting energy expenditure; TEE, total energy expenditure

Detailed information on reported REE and TEE samples, comparison with control cohorts, and generated predictive equations can be found in Supplemental File 2, stratified by disease categories. In the majority of studies (99%; *k* = 267), investigation of energy expenditure was included in the primary research question. Most studies measured REE samples only (*k* = 222, 82%), whereas 22 (8%) measured TEE samples and 27 (10%) measured REE and TEE samples. Figure 2 illustrates the number and type of energy expenditure samples (i.e., REE compared with TEE) conducted across the disease categories. Seven of the 24 disease categories measured REE only (craniopharyngioma, diabetes mellitus, eating disorder, endocrine, gastrointestinal, rheumatic, and other).

### Predictive equations

Very few predictive equations were generated from the energy expenditure data obtained across both REE and TEE (*k* = 18). Energy expenditure predictive equations were generated for cerebral palsy (*k* = 2 [30,31]), cystic fibrosis (*k* = 2 [32,33]), developmental disability (*k* = 2 [32,34]), eating disorders (*k* = 2 [35,36]), gastrointestinal disorders (*k* = 1 [37]), IBD (*k* = 1 [38]), liver disease (*k* = 1 [21]), neurological disorders (*k* = 2 [32,39]), neuromuscular disease (*k* = 1 [22]), Prader-Willi syndrome (*k* = 2 [40,41]), and sickle cell anemia (*k* = 2 [42,43]). Sample sizes of studies that published predictive equations ranged from 5 to 122 [22, 30].

### REE and TEE values compared with controls

A summary of REE and TEE samples compared with healthy controls is detailed in Table 3. Approximately half of REE and TEE samples were compared with a control group (129 [46%] of REE samples and 29 [56%] of TEE samples). In REE samples, craniopharyngioma (86%), diabetes mellitus (83%), and renal (67%) had the most comparisons to control cohorts whereas dermatologic conditions (17%), HIV (17%), and other (0%) had the least. There was variability in the difference in energy expenditure between children with chronic disease compared with control cohorts across and within disease groups.

**TABLE 3:** Description of number of longitudinal studies and studies reporting statistical significance to control groups where available for resting energy expenditure and total energy expenditure in children with chronic disease, stratified by disease group

Disease category	REE						TEE					
	*k*	Longitudinal samples *k* (%)	Control comparison^1^*k* (%)	REE in disease group vs. control			*k*	Longitudinal samples *k* (%)	Control comparison^1^*k* (%)	TEE in disease group vs. control		
				Sig. lower *k* (%)	Sig. higher *k* (%)	No difference *k* (%)				Sig. lower k (%)	Sig. higher k (%)	No difference k (%)
Cancer	19	6 (32)	8 (42)	4 (21)	0 (0)	4 (21)	3	0 (0)	1 (33)	1 (33)	0 (0)	0 (0)
Cerebral palsy	17	2 (12)	8 (47)	5 (29)	1 (6)	2 (12)	7	0 (0)	5 (71)	5 (71)	0 (0)	0 (0)
Congenital heart disease	11	1 (9)	7 (64)^2^	0 (0)	1 (9)	5 (45)	8	3 (38)	7 (88)	0 (0)	4 (50)	3 (38)
Craniopharyngioma	7	1 (14)	6 (86)^2^	4 (57)	0 (0)	1 (14)	0	—	—	—	—	—
Cystic fibrosis	44	9 (20)	24 (55)^3^	0 (0)	12 (27)	9 (20)	10	2 (20)	4 (40)	0 (0)	2 (20)	2 (20)
Dermatologic conditions	6	2 (33)	1 (17)	0 (0)	0 (0)	1 (17)	1	0 (0)	0 (0)	0 (0)	0 (0)	0 (0)
Neurodevelopmental disability	5	1 (20)	2 (40)	0 (0)	1 (20)	1 (20)	4	0 (0)	0 (0)	0 (0)	0 (0)	0 (0)
Diabetes mellitus	6	0 (0)	5 (83)	0 (0)	1 (17)	3 (50)	0	—	—	—	—	—
Eating disorders	10	2 (20)	5 (50)	5 (50)	0 (0)	0 (0)	0	—	—	—	—	—
Endocrine (other)	12	2 (17)	5 (42)	5 (42)	0 (0)	0 (0)	0	—	—	—	—	—
Gastrointestinal (other)	3	0 (0)	1 (33)	0 (0)	0 (0)	1 (33)	0	—	—	—	—	—
Genetic disorders (other)	12	3 (25)	7 (58)^2^	4 (33)	0 (0)	2 (17)	4	0 (0)	3 (75)^5^	1 (25)	0 (0)	1 (25)
HIV	6	0 (0)	1 (17)	0 (0)	0 (0)	1 (17)	2	0 (0)	0 (0)	0 (0)	0 (0)	0 (0)
IBD	17	5 (29)	5 (29)	1 (6)	2 (12)	2 (12)	1	1 (100)	1 (100)	1 (100)	0 (0)	0 (0)
Liver disease	8	0 (0)	4 (50)	0 (0)	3 (38)	1 (13)	1	0 (0)	1 (100)	0 (0)	1 (100)	0 (0)
Metabolic disorders	13	2 (15)	3 (23)	0 (0)	1 (8)	2 (15)	1	1 (100)	0 (0)	0 (0)	0 (0)	0 (0)
Neurologic (other)	14	1 (7)	6 (43)	4 (29)	0 (0)	2 (14)	2	0 (0)	2 (100)	2 (100)	0 (0)	0 (0)
Neuromuscular diseases	21	2 (10)	7 (33)^4^	4 (19)	1 (5)	1 (5)	1	0 (0)	0 (0)	0 (0)	0 (0)	0 (0)
Prader-Willi syndrome	12	6 (50)	3 (25)	2 (17)	0 (0)	1 (8)	4	1 (25)	2 (50)	1 (25)	0 (0)	1 (25)
Renal	6	0 (0)	4 (67)^2^	1 (17)	1 (17)	1 (17)	1	0 (0)	1 (100)	0 (0)	1 (100)	0 (0)
Respiratory (other)	7	0 (0)	3 (43)	1 (14)	1 (14)	1 (14)	1	0 (0)	1 (100)	0 (0)	0 (0)	1 (100)
Rheumatic	3	0 (0)	1 (33)	0 (0)	0 (0)	1 (33)	0	—	—	—	—	—
Sickle cell anemia	20	0 (0)	13 (65)^2^	0 (0)	12 (60)	0 (0)	1	0 (0)	1 (100)	0 (0)	0 (0)	1 (100)
Other	4	0 (0)	0 (0)	0 (0)	0 (0)	0 (0)	0	—	—	—	—	—
Total	**283**	**45 (16)**	**129 (46)**	**40 (14)**	**37 (13)**	**42 (15)**	**52**	**8 (15)**	**29 (56)**	**11 (21)**	**8 (15)**	**9 (17)**

Abbreviations: HIV, human immunodeficiency virus; IBD, inflammatory bowel disease; *k*, number of studies; REE, resting energy expenditure; sig., statistically significantly; TEE, total energy expenditure. Percentages calculated as percent of total REE and TEE samples, respectively.

1 Controls represent children without identified diseases, i.e., healthy controls.

2 1× REE disease vs. control comparison not reported.

3 3× REE disease vs. control comparison not reported.

4 1 study – REE higher than controls but no statistical comparison.

5 1× REE disease vs. control comparison not reported.

### Quality assessment

The Porter et al. [20] quality assessment revealed significant variability in the reporting of the methodology relating to measurement of REE and TEE samples (Table 4). Only 5 (2%) studies reported that the minimal conditions for resting metabolic rate data collection were met. Adequate fasting periods were reported for most studies measuring REE (65%), whereas abstinence of physical activity (13%), thermal neutral environment (29%) and appropriate acclimation protocols (27%) were mostly not met and/or not reported. The method of IC and DLW data collection (machine or type of canopy [85%], 2-point or multipoint [98%], dose [86%], and conversion method [73%]) were well reported. Comparatively, detailed information regarding obtaining acceptable data (26%) and the precision of both REE (13%) and TEE (14%) samples were lacking.

**TABLE 4:** Quality assessment of the measurement of resting energy expenditure and total energy expenditure completed by included studies utilizing Porter et al. [20] Quality Assessment Tool

Quality component	Yes, *n* (%)
REE (*k* = 249 studies)	
Part A: Were the minimal conditions for the measurement of RMR met?	
1. Fasting period of a minimum 5 h before measurement?	161 (65)
2. Abstinence of moderate physical activity for ≥2 h or vigorous physical activity for ≥14 h before measurement?	33 (13)
3. Were the environmental conditions thermal neutral (room temp of 20°C–25°C, 68°F–77°F)?	71 (29)
4. Did the subjects lie down in a quiet room for ≥15–30 min before the measurement began and acclimate for 3–5 min before the measurement and were ≥5 min of steady state respiratory gas exchange data collected?	68 (27)
Part B: Was the methodology fully reported?	
1. Were the conditions listed under Part A reported?	5 (2)
2. Was information about obtainment of acceptable data for analysis reported?	64 (26)
3. Was the method of Indirect Calorimetry stated? (e.g., name of machine and/or use of mask/canopy/Douglas bag)	212 (85)
4. Was the precision of the measurement reported?	32 (13)
TEE (*k* = 49 studies)	
1. Was the method for DLW stated (e.g. 2-point or multipoint) or could it be clearly deduced from the methods?	48 (98)
2. Was the dose and method of calculating DLW dose clearly stated?	42 (86)
3. Was the method for converting cabon dioxide production to energy expenditure reported?	36 (73)
4. Did the participants fast for 5–12 h before dosing?	11 (22)
5. Did the isotope ratio analyses meet the precision requirements for 5%–10% precision of total energy expenditure?	7 (14)

Abbreviations: DLW, doubly labeled water; *k*, number of studies; REE, resting energy expenditure; RMR, resting metabolic rate; TEE, total energy expenditure.

## Discussion

This systematic review comprehensively summarizes the existing literature on energy expenditure in children with chronic diseases and identifies several gaps in the evidence base. There is currently a large and diverse evidence base, primarily focusing on the diseases that significantly impact nutritional outcomes such as cystic fibrosis. A greater emphasis was placed on measuring REE rather than that of TEE, with very few exploring both samples within the same study. Over 60% of retrieved studies were published >10 y ago, and studies were generally conducted with relatively small sample sizes. Significant limitations were also identified in how the samples of energy expenditure were reported.

Mapping of the retrieved evidence by disease category revealed that only a few diseases were explored in depth; only cystic fibrosis, cancer, cerebral palsy, sickle cell disease, and neuromuscular disease had 20 or more single samples of energy expenditure. These conditions may be particularly well-studied due to the recognized and significant nutritional impacts that they have. Additionally, children with these chronic conditions typically present with altered body composition such as reduced lean muscle mass, a key determinant of energy expenditure [2]. In contrast, despite well-described nutritional effects in adulthood [[44], [45], [46]], there were several chronic conditions that had minimal numbers of disease groups studied in pediatric populations such as liver disease (*k* = 8), respiratory (*k* = 8), and HIV (*k* = 6). It is well documented that children with liver disease become very malnourished; however, potentially due to its rarity, the critical nature of the condition, and frequency of medical interventions and surgeries, this population may be limited in its capacity to be well-studied [47]. Similarly, HIV tends to be more prominent in children in developing countries, where access to energy expenditure measurement may be limited or may not be a priority for study within a resource-poor environment [46]. Hence, the differences between disease groups that were and were not well studied may be attributed to difficulties in obtaining and/or a lack of perceived need for this data.

Included studies predominantly focused on REE, with only 9% of studies measuring TEE, and a similar proportion (10%) reporting on both REE and TEE samples. However, this is not entirely unexpected. Compared to REE, measuring TEE using DLW increases the complexity and feasibility of the study. Specifically, the study duration is often extended beyond 1 visit to accommodate biologic sample collection, which increases the burden on children and families; sample collection can be challenging in young children or children with significant physical disability; the costs of purchasing DLW (∼$1000 Australian Dollar per dose) and sample analysis are often beyond the budgetary constraints of clinical research projects; and specific expertise is required to complete the analysis of samples via MS [48]. The costly nature of measuring TEE may particularly be a barrier for rare diseases for which research funding is limited. These challenges demonstrate the need for validation of less costly and more practical devices against DLW to measure TEE. Additionally, the major advantage of investigating REE and TEE data concurrently is that it enables energy expended in physical activity to be derived. This would be particularly beneficial for chronic diseases that can limit physical activity levels such as cerebral palsy, neuromuscular diseases, and Prader-Willi syndrome [49].

In some chronic conditions, the retrieved evidence may no longer be relevant to guide energy expenditure prescription due to advances in care, particularly as over two-thirds of the included studies were published >10 y ago. Disease-modifying therapies for many chronic conditions are emerging that have the potential to change the natural history of a disease and consequently alter or even normalize energy expenditure. For example, in sickle cell anemia, the most recent literature on energy expenditure was published in 2007; however, gene editing and pharmacologic therapies for the disease have been introduced since that study [50]. In cystic fibrosis, the Food and Drug Administration recently approved a modulator therapy including elexacaftor, tezacaftor, or ivacaftor, which has demonstrated promising results in improving lung function and reducing pulmonary exacerbation [51]. Given the known effect of pulmonary function on energy expenditure, there is potential for observable changes in energy expenditure in children with cystic fibrosis treated with similar therapies. Another example is the emergence of disease-modifying therapies for spinal muscular atrophy (SMA), which was the most common genetic cause of infant mortality [52]. There are now 3 approved therapies for SMA: nusinersen, risdiplam, and onasemnogene abeparvovec; if initiated in presymptomatic infants, these therapies may result in almost normal motor development [52]. Interestingly, 2 studies included in this review measured REE in children with SMA. One study demonstrated that nusinersen treatment was associated with a higher REE [52], whereas the second study showed no difference in measured compared with estimated REE using Schofield and SMA-specific equations in a nusinersen-treated cohort [53]. For children with achondroplasia, the most common form of disproportionate short stature [54], there were no studies identified in this review that measured energy expenditure. There are now therapies available for achondroplasia that increase linear growth in children [55] and as such, there is a need for further research exploring energy expenditure, in particular the energy cost of increased growth. The increasing availability of precision treatments and improved management for pediatric conditions highlight the need for continually updated contemporary research on measured energy expenditure in children with chronic disease. With updated research in energy requirements, there is an opportunity to provide precision nutrition therapy for children with chronic disease. Without updated research, there is risk of nutritional mismanagement and malnutrition in vulnerable populations.

Most studies included in this review utilized observational study designs. Use of well-designed observational studies, such as prospective cohort studies, will provide the opportunity to document energy expenditure over time in comparison to control groups. Comparisons with untreated disease controls also enable identification of the effect of disease-modifying therapies on energy expenditure on specific chronic diseases. Additionally, the majority of the studies had <50 participants. This highlights the challenge in recruiting large sample sizes of children with chronic disease, particularly those with rare disorders such as endocrine conditions and metabolic disorders. Small sample sizes also decrease statistical power, reducing the capacity to identify differences in energy expenditure in children without chronic disease or in other disease controls. The small sample sizes may have contributed to the lack of studies that generated energy expenditure equations. Some of the most clinically used predictive energy expenditure equations for healthy pediatric populations are Harris and Benedict [56], FAO/WHO/UNU [57], and Schofield [58]. The latter 2 were developed with particularly large sample sizes, utilizing international populations to obtain sample sizes of *n* = 7000 and *n* = 2359, respectively [59]. Additionally, FAO/WHO/UNU and Schofield were evaluated to have the highest *R*^2^ values compared with other equations to estimate energy expenditure in pediatric populations [59]. This further emphasizes the importance of adequate sample size to determine accurate equations. There is a need to identify more effective methods to obtain energy expenditure data collectively, potentially requiring collaboration nationally or internationally to provide greater sample sizes to generate predictive equations to guide clinical management of children with chronic diseases.

Differences in energy expenditure in children with chronic disease compared with control was observed for the majority of the disease categories identified, demonstrating the need for accurate estimations of energy expenditure in children with chronic conditions. The variability in energy expenditure in chronic diseases compared with control cohorts is likely due to differences in the characteristics of the control groups. Studies used a mixture of different healthy controls, matched according to different characteristics such as age, weight, body composition, and/or pubertal development. Although meta-analysis of energy expenditure samples in disease groups compared with controls was not completed, this review provides a key starting point to enable clinicians to drive future nutrition research agendas in disease groups.

Overall, the quality assessment of energy expenditure samples was poor. It is unclear as to whether it is due to lack of adherence to minimal conditions required and/or the lack of reporting in studies. This uncertainty influences the validity and reliability of the energy expenditure samples in the included studies and highlights how poorly described methods of energy expenditure measurement hinder the ability to accurately assess quality and compare outcomes across studies. The JBI tool is a well-accepted tool for quality assessment, utilizing study design specific questions to appraise possibility of bias in its design, conduct, and analysis [60]. Although it was completed for all included studies (Supplemental File 2), it was not included in the final analysis. The JBI tool asks the question of whether the method used to obtain the data was valid and reliable. Due to the inclusion criteria of the current review, all studies would have met this condition, in which energy expenditure samples were required to have been obtained either through gold-standard measurements of IC or DLW. However, the Porter et al. [20] quality assessment tool, which incorporates energy expenditure-specific quality questions, revealed that despite utilizing gold-standard measurements, there was unclear reporting and/or poor adherence to meeting minimal conditions for the obtainment of this data. This demonstrates the difficulty of using quality assessment tools that are generic to study design and highlights the need for standardized reporting protocols based on energy expenditure-specific quality assessment tools. Proper instrumentation and adherence to measurement protocols will enable more meaningful assessments of nutritional status [61].

This systematic review has several strengths. Despite being in the inclusion criteria, no systematic reviews or meta-analyses that address this review question were identified during screening. This indicates that there is currently an absence of studies collating REE or TEE data in children across all chronic diseases identified. Therefore, this review contributes to filling this specific gap in the scientific literature and provides a reference for any future research in this topic. Additionally, the formal process derived from the “PRISMA recommended guidelines” was utilized [16]. Although a comprehensive literature search utilizing well-defined terms and inclusion or exclusion criteria was conducted to ensure all relevant studies were included, there may be some rare pediatric chronic conditions that were not captured. Also, due to the breadth of the search, the number of studies obtained and consequently the number of chronic diseases explored was large, and values of REE and TEE were unable to be further synthesized. Consequently, known influences on energy expenditure such as lean muscle mass, age, and their potential mechanisms on energy expenditure in children with chronic disease were unable to be explored.

### Future research priorities

This review has revealed several opportunities for future research. Greater primary studies with larger sample sizes should be conducted in diseases where studies were lacking. Additionally, the impact of advances in disease management (e.g., medications, treatment therapies) on energy expenditure should be researched. The data provided in Supplemental File 2 can facilitate studies or individual-level systematic reviews and meta-analyses to be conducted in disease groups. Aggregated data analysis can demonstrate differences in energy expenditure between children with chronic diseases and their controls and explore how factors such as body composition impact these relationships. Aggregated datasets may also be able to generate disease-specific predictive equations. Beyond updating the current literature, key challenges in the study quality highlight the need for better reporting of IC and TEE protocols and emphasizes the need for validation of less costly and more practical devices against DLW to measure TEE.

### Conclusion

This systematic review is the first, to our knowledge, to comprehensively synthesize and summarize the available evidence on energy expenditure samples using gold-standard techniques in children with chronic diseases. The literature revealed that the majority of studies were conducted on cystic fibrosis, cancer, and sickle cell anemia, measuring REE, with fewer studies measuring TEE using DLW. Several future research priorities have been highlighted to provide a clearer understanding of energy expenditure in children, which will help guide the nutritional management of children with chronic diseases. International collaboration and more robust measurement and/or reporting of energy expenditure should be conducted to generate meaningful predictive energy equations to provide an updated literature base that is reflective of the emerging disease-modifying therapies.

## Author contributions

The authors’ responsibilities were as follows—conception and design of the research: ZED, EV, KN; data acquisition, analysis, and interpretation: BL, ZED, KO, EV, JL, KD, ML, NB, KN; drafting of the manuscript: BL, ZED; critical revision of the manuscript: BL, ZED, KO, EV, JL, KD, ML, NB, KN; and all authors: read and approved the final manuscript.

## Conflict of interest

The authors report no conflicts of interest.

## Funding

The authors reported no funding received for this study.

## Data availability

The authors confirm that the data supporting the findings of this study are available within the article and its supplementary materials.
