# Supplementary material for: Describing Energy Expenditure in Children with a Chronic Disease: A Systematic Review
Source: Adv Nutr. 2024 Mar 1;15(4):100198. doi: 10.1016/j.advnut.2024.100198 (PMC10987846; doi:10.1016/j.advnut.2024.100198)
Supplement: Multimedia component 1 [file mmc1.docx]

**Search Strategies for Databases OVID MedLine, Embase, CINAHL Plus, Cochrane and Scopus**

**OVID MedLine Search Strategy**

| Part A – Children and Adolescents (Population) | |
| --- | --- |
| 1 | exp Child/ |
| 2 | exp Adolescent/ |
| 3 | Exp Infant/ |
| 4 | Child*.mp. |
| 5 | adolescen*.mp. |
| 6 | p?ediatric*.mp. |
| 7 | teen*.mp. |
| 8 | youth*.mp. |
| 9 | 1 or 2 or 3 or 4 or 5 or 6 or 7 or 8 |
| Part B – Chronic Diseases and Conditions (Intervention) | |
| 10 | exp Chronic Disease/ |
| 11 | chronic*.mp. |
| 12 | disease*.mp. |
| 13 | long term.mp. |
| 14 | chronic* ill*.mp. |
| 15 | cancer.mp. |
| 16 | diabetes.mp. |
| 17 | cystic fibrosis.mp. |
| 18 | Cystic Fibrosis/ |
| 19 | dystrophy.mp. |
| 20 | Pulmonary Disease, Chronic Obstructive/ |
| 21 | renal insufficiency, chronic/ |
| 22 | kidney failure, chronic/ |
| 23 | syndrome*.mp. |
| 24 | Syndrome/ |
| 25 | oncolog*.mp. |
| 26 | exp Medical Oncology/ |
| 27 | an?emia.mp. |
| 28 | autoimmune.mp. |
| 29 | immune*.mp. |
| 30 | disorder.mp. |
| 31 | inflamma*.mp. |
| 32 | neuro*.mp. |
| 33 | cardiovascular.mp. |
| 34 | exp Cardiovascular Diseases/ |
| 35 | Coronary Disease/ |
| 36 | arthritis.mp. |
| 37 | osteo*.mp. |
| 38 | disabilit*.mp. |
| 39 | cerebral palsy.mp. |
| 40 | Cerebral Palsy/ |
| 41 | spinal muscular atrophy.mp. |
| 42 | neuromuscular disorder.mp. |
| 43 | dystrophin*.mp. |
| 44 | Muscular Dystrophy, Duchenne/ |
| 45 | Muscular Atrophy, Spinal/ |
| 46 | Infant, Premature |
| 47 | 10 or 11 or 12 or 13 or 14 or 15 or 16 or 17 or 18 or 19 or 20 or 21 or 22 or 23 or 24 or 25 or 26 or 27 or 28 or 29 or 30 or 31 or 32 or 33 or 34 or 35 or 36 or 37 or 38 or 40 or 41 or 42 or 43 or 44 or 45) not 46 |
| Part C – Measures of Energy Expenditure (Exposure) | |
| 48 | energy requirement*.mp. |
| 49 | energy expenditure*.mp. |
| 50 | total energy expenditure.mp. |
| 51 | resting energy expenditure.mp. |
| 52 | basal metabolic rate.ti,ab |
| 53 | Basal Metabolism/ |
| 54 | Calorimetry, Indirect/ |
| 55 | 48 or 49 or 50 or 51 or 52 or 53 or 54 |
| Part A AND Part B AND Part C | |
| 56 | 9 and 47 and 55 |
| English language and human only | |
| 57 | limit 56 to (English language and humans) |
| Search Update | |
| 58 | limit 57 to yr=“2020-2022” |

**Embase**

| Part A – Children and Adolescents (Population) | |
| --- | --- |
| 1 | exp Child/ |
| 2 | exp Adolescent/ |
| 3 | Exp Infant/ |
| 4 | Child*.mp. |
| 5 | adolescen*.mp. |
| 6 | p?ediatric*.mp. |
| 7 | teen*.mp. |
| 8 | youth*.mp. |
| 9 | 1 or 2 or 3 or 4 or 5 or 6 or 7 or 8 |
| Part B – Chronic Diseases and Conditions (Intervention) | |
| 10 | exp Chronic Disease/ |
| 11 | chronic*.mp. |
| 12 | disease*.mp. |
| 13 | long term.mp. |
| 14 | chronic* ill*.mp. |
| 15 | cancer.mp |
| 16 | Exp diabetes mellitus/ |
| 17 | Exp cystic fibrosis/ |
| 18 | Exp dystrophy/ |
| 19 | Chronic obstructive pulmonary dis*.mp. |
| 20 | Exp chronic kidney failure/ |
| 21 | Exp Syndrome/ |
| 22 | oncolog*.mp. |
| 23 | exp Oncology/ |
| 24 | an?emia.mp. |
| 25 | autoimmun*.mp. |
| 26 | immune*.mp. |
| 27 | Disorder*.mp. |
| 28 | inflamma*.mp. |
| 29 | neuro*.mp. |
| 30 | exp Cardiovascular Diseases/ |
| 31 | Exp Coronary artery Disease/ |
| 32 | Exp arthritis/ |
| 33 | osteo*.mp. |
| 34 | disabilit*.mp. |
| 35 | Exp cerebral palsy/ |
| 36 | Exp spinal muscular atrophy/ |
| 37 | Exp neuromuscular disease/ |
| 38 | dystrophin*.mp. |
| 39 | Exp Duchenne Muscular Dystrophy/ |
| 40 | Exp Spinal Muscular Atrophy/ |
| 41 | prematur* infan* |
| 42 | 10 or 11 or 12 or 13 or 14 or 15 or 16 or 17 or 18 or 19 or 20 or 21 or 22 or 23 or 24 or 25 or 26 or 27 or 28 or 29 or 30 or 31 or 32 or 33 or 34 or 35 or 36 or 37 or 38 or 40) not 41 |
| Part C – Measures of Energy Expenditure (Exposure) | |
| 43 | energy requirement*.mp. |
| 44 | Exp energy expenditure/ |
| 45 | total energy expenditure.mp. |
| 46 | resting energy expenditure.mp. |
| 47 | Exp basal metabolic rate/ |
| 48 | Basal Metabol*.mp. |
| 49 | Exp Indirect Calorimetry/ |
| 50 | 43 or 44 or 45 or 45 or 46 or 47 or 48 or 49 or 50 |
| Part A AND Part B AND Part C | |
| 51 | 9 and 42 and 50 |
| English language and human only | |
| 52 | limit 51 to (English language and humans) |
| Search Update | |
| 53 | limit 52 to yr=“2020-2022” |

**CINAHL Plus**

| Part A – Children and Adolescents (Population) | |
| --- | --- |
| 1 | (MH "Child+") |
| 2 | (MH "Adolescence+") |
| 3 | (MH "Infant+") |
| 4 | Child* |
| 5 | adolescen* |
| 6 | P#ediatric* |
| 7 | teen* |
| 8 | youth* |
| 9 | (S1 OR S2 OR S3 OR S4 OR S5 OR S6 OR S7 OR S8) |
| Part B – Chronic Diseases and Conditions (Intervention) | |
| 10 | (MH "Chronic Disease+") |
| 11 | chronic* disease* |
| 12 | chronic* ill* |
| 13 | cancer |
| 14 | (MH "Diabetes Mellitus+") |
| 15 | (MH "Cystic Fibrosis") |
| 16 | Dystroph* |
| 17 | (MH "Pulmonary Disease, Chronic Obstructive+") |
| 18 | (MH "Kidney Failure, Chronic+") OR (MH "Renal Insufficiency+") OR (MH "Renal Insufficiency, Chronic+") |
| 19 | (MH "Oncology+") |
| 20 | (MH "Anemia+") |
| 21 | An#emi* |
| 22 | (MH "Autoimmune Diseases+") |
| 23 | Immun* Disorder* |
| 24 | Inflamm* |
| 25 | Neuro* |
| 26 | (MH "Cardiovascular Diseases+") |
| 27 | (MH "Coronary Arteriosclerosis") |
| 28 | (MH "Arthritis+") |
| 29 | osteo* |
| 30 | disabilit* |
| 31 | (MH "Cerebral Palsy") |
| 32 | (MH "Muscular Atrophy, Spinal+") |
| 33 | (MH "Neuromuscular Diseases+") |
| 34 | Dystroph* |
| 35 | (MH "Muscular Dystrophy, Duchenne+") |
| 36 | (MH "Infant, Premature") |
| 37 | S10 OR S11 OR S12 OR S13 OR S14 OR S15 OR S16 OR S17 OR S18 OR S19 OR S20 OR S21 OR S22 OR S23 OR S24 OR S25 OR S26 OR S27 OR S28 OR S29 OR S30 OR S31 OR S32 OR S33 OR S34 OR S35  NOT S36 |
| Part C – Measures of Energy Expenditure (Exposure) | |
| 38 | energy requirement* |
| 39 | (MH "Energy Metabolism+") |
| 40 | “total energy expenditure” |
| 41 | “resting energy expenditure” |
| 42 | (MH "Basal Metabolic Rate") |
| 43 | basal metaboli* |
| 44 | Indirect Calorimet* |
| 45 | 38 or 39 or 40 or 41 or 42 or 43 or 44 |
| Part A AND Part B AND Part C | |
| 56 | 9 and 37 and 45 |
| English language and human only | |
| 57 | limit 56 to (English language and humans) |
| Search Update | |
| 58 | limit 57 to yr=“2020-2022” |

**Cochrane**

Search Name: SLR Energy Expenditure

Last Saved: 04/07/2023 21:35:22

Comment:

ID Search

#1 [mh child]

#2 [mh adolescent]

#3 [mh infant]

#4 child*

#5 adolescen*

#6 p?ediatric*

#7 teen*

#8 youth*

#9 #1 OR #2 OR #3 OR #4 OR #5 OR #6 OR #7 OR #8

#10 [mh "Chronic Disease"]

#11 chronic* OR disease* OR long term OR chronic* ill*

#12 cancer

#13 diabetes

#14 cystic fibrosis

#15 [mh ^"Cystic Fibrosis"]

#16 dystrophy

#17 [mh ^"Pulmonary Disease, Chronic Obstructive"]

#18 [mh ^"renal insufficiency, chronic"]

#19 [mh ^"kidney failure, chronic"]

#20 syndrome*

#21 [mh ^Syndrome]

#22 oncolog*

#23 [mh "Medical Oncology"]

#24 an?emia

#25 autoimmune

#26 immuno*

#27 disorder

#28 inflamma*

#29 neuro*

#30 cardiovascular

#31 [mh ^"Cardiovascular Diseases"]

#32 [mh ^"Coronary Disease"]

#33 arthritis

#34 osteo*

#35 disabilit*

#36 cerebral palsy

#37 [mh ^"Cerebral Palsy"]

#38 spinal muscular atrophy

#39 neuromuscular disorder

#40 dystrophin*

#41 [mh ^"Muscular Dystrophy, Duchenne"]

#42 [mh ^"Muscular Atrophy, Spinal"]

#43 [mh ^"Infant, Premature"]

#44 #10 OR #11 OR #12 OR #13 OR #14 OR #15 OR #16 OR #17 OR #18 OR #19 OR #20 OR #21 OR #22 OR #23 OR #24 OR #25 OR #26 OR #27 OR #28 OR #29 OR #30 OR #31 OR #32 OR #33 OR #34 OR #35 OR #36 OR #37 OR #38 OR #39 OR #40 OR #41 OR #42 NOT #43

#45 energy requirement*

#46 energy expenditure*

#47 total energy expenditure

#48 resting energy expenditure

#49 basal metabolic rate:ti

#50 basal metabolic rate:ab

#51 [mh ^"Basal Metabolism"]

#52 [mh ^"Calorimetry, Indirect"]

#53 #45 OR #46 OR #47 OR #48 OR #49 OR #50 OR #51 OR #52

#54 #9 AND #44 AND #53

#55 [mh animals] NOT [mh humans]

#56 #54 NOT #55 with Cochrane Library publication date Between Dec 2021 and Jul 2023

**Scopus**

( ( TITLE-ABS-KEY ( child* ) ) OR ( TITLE-ABS-KEY ( adolescen* ) ) OR ( TITLE-ABS-KEY ( infan* ) ) OR ( TITLE-ABS-KEY ( paediatric* ) ) OR ( TITLE-ABS-KEY ( pediatric* ) ) OR ( TITLE-ABS-KEY ( teen* ) ) OR ( TITLE-ABS-KEY ( youth* ) ) ) AND ( ( ( TITLE-ABS-KEY ( chronic* ) ) OR ( TITLE-ABS-KEY ( disease* ) ) OR ( TITLE-ABS-KEY ( "chronic disease*" ) ) OR ( TITLE-ABS-KEY ( "long term" ) ) OR ( TITLE-ABS-KEY ( "chronic* ill*" ) ) OR ( TITLE-ABS-KEY ( cancer* ) ) OR ( TITLE-ABS-KEY ( diabetes* ) ) OR ( TITLE-ABS-KEY ( "cystic fibrosis" ) ) OR ( TITLE-ABS-KEY ( dystroph* ) ) OR ( TITLE-ABS-KEY ( "chronic obstruction pulmonary disease" ) ) OR ( TITLE-ABS-KEY ( "chronic kidney failure" ) ) OR ( TITLE-ABS-KEY ( "chronic renal insufficienc*" ) ) OR ( TITLE-ABS-KEY ( syndrome* ) ) OR ( TITLE-ABS-KEY ( oncolog* ) ) OR ( TITLE-ABS-KEY ( anaemi* ) ) OR ( TITLE-ABS-KEY ( anemi* ) ) ) OR ( ( TITLE-ABS-KEY ( autoimmun* ) ) OR ( TITLE-ABS-KEY ( immun* ) ) OR ( TITLE-ABS-KEY ( disorder* ) ) OR ( TITLE-ABS-KEY ( inflamma* ) ) OR ( TITLE-ABS-KEY ( neuro* ) ) OR ( TITLE-ABS-KEY ( cardiovascular ) ) OR ( TITLE-ABS-KEY ( "coronary disease" ) ) OR ( TITLE-ABS-KEY ( arthritis ) ) OR ( TITLE-ABS-KEY ( osteo* ) ) OR ( TITLE-ABS-KEY ( disabilit* ) ) OR ( TITLE-ABS-KEY ( "cerebral palsy" ) ) OR ( TITLE-ABS-KEY ( "spinal muscular atrophy" ) ) OR ( TITLE-ABS-KEY ( "neuromuscular disorder*" ) ) OR ( TITLE-ABS-KEY ( dystrophin* ) ) OR ( TITLE-ABS-KEY ( "duchenne muscular dystroph*" ) ) ) AND NOT ( TITLE-ABS-KEY ( "premature infan*" ) ) ) AND ( ( TITLE-ABS-KEY ( "energy requirement*" ) ) OR ( TITLE-ABS-KEY ( "energy expenditure*" ) ) OR ( TITLE-ABS-KEY ( "total energy expenditure*" ) ) OR ( TITLE-ABS-KEY ( "resting energy expenditure*" ) ) OR ( TITLE-ABS-KEY ( "basal metabolic rate*" ) ) OR ( TITLE-ABS-KEY ( "basal metaboli*" ) ) OR ( TITLE-ABS-KEY ( "indirect calorimetry" ) ) ) AND ( LIMIT-TO ( PUBYEAR , 2021 ) OR LIMIT-TO ( PUBYEAR , 2022 ) OR LIMIT-TO ( PUBYEAR , 2023 ) )
